# Comparison of Non-invasive Liver Fat Scoring Systems as Markers of Metabolic Dysfunction-Associated Liver Disease

**DOI:** 10.7759/cureus.72222

**Published:** 2024-10-23

**Authors:** Eunice S Thomson, Akash T Oommen, Sheejamol V S, Gopalakrishna Pillai

**Affiliations:** 1 Internal Medicine, Amrita Institute of Medical Sciences and Research Centre, Kochi, IND; 2 Biostatistics, Amrita Institute of Medical Sciences and Research Centre, Kochi, IND

**Keywords:** dyslipidemia, fatty liver index, hepatic steatosis (masld), metabolic syndrome (mets), nafld liver fat score, nonalcoholic fatty liver disease (nafld), obesity, systemic hypertension, type-2 diabetes mellitus, visceral adiposity index

## Abstract

Background

Metabolism dysfunction-associated steatotic liver disease (MASLD) is hepatic steatosis along with increased weight or obesity, type 2 diabetes, or metabolic dysregulation and without significant alcohol consumption. The clinical prediction of MASLD using simple, non-invasive indices like Fatty Liver Index (FLI), NAFLD Liver Fat Score (NAFLD LFS), Visceral Adiposity Index (VAI), Steato Test and Hepatic Steatosis Index (HSI) yielded heterogeneous results in different populations.

Aim

We aimed to compare five scores (Fatty Liver Index, NAFLD Liver Fat Score, Visceral Adiposity Index, Steato Test, Hepatic Steatosis Index) for prediction of liver steatosis.

Method

Patients with metabolic syndrome and alcohol intake less than 20 grams per day were included in the study. Patients with alcohol intake greater than 20 grams per day and known history of liver or biliary disease, infections, muscle injury, autoimmune diseases, thyroid disorders, underlying secondary diabetes mellitus, secondary hypertension, familial dyslipidemia, intake of medications like steroids, hepatotoxic drugs, chemotherapy or drugs altering liver function tests were excluded. The presence of fatty liver was confirmed on ultrasound. Five indices (Fatty Liver Index, NAFLD Liver Fat Score, Visceral Adiposity Index, Steato Test, Hepatic Steatosis Index) were applied to predict liver steatosis. The correlation of each index with presence of fatty liver was analyzed. Based on the sensitivity of the five scoring systems and prevalence of MASLD as observed in existing literature, with a 95% confidence interval and 20% allowable error, the minimum number of positive cases required is 24 and minimum sample size required is 77.

Results

Among 100 (100%) patients with metabolic syndrome, MASLD was seen in 65 patients (74% males, 56% females). The mean age of patients with MASLD was 59 years. Fatty Liver Index, NAFLD Liver Fat Score, Visceral Adiposity Index, Steato Test had statistically significant (p<0.005) correlation with fatty liver on ultrasound of abdomen. Fatty Liver Index had the highest Area Under the Receiver Operating Characteristic curve (AUROC) of 0.65 (Sensitivity=63, Specificity=62.9%), followed by NAFLD Liver Fat Score (AUROC=0.63, Sensitivity=64.4%, Specificity=62.9%), Visceral Adiposity Index (AUROC=0.628, Sensitivity=50.8%, Specificity=65.7%) and Steato Test (AUROC=0.61, Sensitivity=46.2%, Specificity=77%). Cut-offs for Fatty Liver Index, NAFLD Liver Fat Score, Visceral Adiposity Index, and Steato Test were 42, 0.5, 4, and 4.5 respectively. MASLD was present in 71.4% (N=35) of patients with type 2 diabetes mellitus and 58.8% (N=30) without type 2 diabetes mellitus.

Conclusion

Fatty Liver Index, NAFLD Liver Fat Score, Visceral Adiposity Index, and Steato Test were comparable as early markers to predict liver steatosis in patients with metabolic syndrome.

## Introduction

In the presence of histological or radiological proof of hepatic steatosis and no other reason for the accumulation of secondary hepatic fat, such as heavy alcohol use, specific drug usage, hereditary diseases, or other conditions, the patient could be then diagnosed with metabolic dysfunction-associated steatotic liver disease (MASLD) [1]. The accumulation of fat within the liver cells is called hepatic steatosis. In MASLD, the distribution of fat is characterized by macrovesicles, which lead to mitochondrial dysfunction and enhanced reactive oxygen-free radical production. Inflammation, cell death, and fibrosis are the outcomes of this process [2]. Metabolic syndrome (MetS) is comprised of the following four metabolic disorders: systemic hypertension (HTN), dyslipidemia, insulin resistance or type 2 diabetes mellitus (type 2 DM), and truncal obesity, also referred to as the deadly quartet [3]. MetS and MASLD go hand in hand. The rate of MetS among Asian Indians varies by area, lifestyle, socioeconomic status, and degree of urbanization [4].

When it comes to MASLD, ultrasonography is the go-to diagnostic method because of its inexpensive cost and widespread availability. On ultrasound, the bright score is used to categorize fatty liver disease into Mild, Moderate and Severe. This is using several parameters like brightness of the parenchyma, contrast of liver in comparison to the kidney, deep beam attenuation, bright vessel walls, and definition of the gallbladder wall [5]. The most reliable way to determine the extent of liver involvement, however, is by liver biopsy [6], but this invasive technique is not commonly performed because of the risks associated with it. There are several scoring systems for the prediction of MASLD, which can help the physician predict MASLD earlier.

When it comes to national programs, India is among the pioneers in addressing fatty liver disease [7]. People are more likely to make healthier lifestyle choices, such as starting an exercise routine, eating well, and controlling related conditions, when educated regarding the illness. Management is a cumbersome process and requires cooperation from the patient’s side. The mainstay is dietary restriction and exercise. Limiting the intake of advanced glycation end products (AGEs) by reducing foods rich in protein, fat, baked and grilled foods. Research has shown a beneficial effect with use of vinegar [8] and has suggested a positive impact with vitamin E supplementation [9]. Omega-3 supplementation has advantageous benefits on non-alcoholic fatty liver disease (NAFLD). A 12-week period of taking omega-3 supplements at a dosage of 2 g per day led to a decrease in visceral fat and an enhancement in the score of fatty liver [10]. Randomized control trials have shown that fecal microbiota transplant (FMT) can attenuate fatty liver disease by improving gut microbiota [11]. It is also essential to control the associated type 2 DM, hypertension and dyslipidemia, by maintaining normal ranges of hemoglobin A1c (HbA1c) and fasting lipid parameters, to prevent development of other complications. This study used five scoring systems to evaluate which will correctly predict fatty liver disease in patients with metabolic syndrome. An early diagnosis of fatty liver disease will help the individuals make a more meaningful lifestyle change and prevent the progression of the illness. 

## Materials and methods

Study design

This study was conducted as a single center, prospective, diagnostic study over a period of six months from August 2023 to January 2024 in a tertiary care hospital in South India. The study was approved by the Ethics Committee of Amrita School of Medicine (approval ECASM-AIMS-2023-079).

Inclusion and exclusion criteria

Patients who were above the age of 18 years and had MetS were included in the study.

Patients who were excluded from the study were those who were known to have an alcohol intake greater than 20 grams per day, known history of liver or biliary disease (including chronic liver disease), infections, muscle injury, autoimmune diseases, thyroid disorders, underlying secondary diabetes mellitus, secondary hypertension, familial dyslipidemia, intake of medications like steroids, hepatotoxic drugs, chemotherapy or dugs altering liver function tests.

Sample size

Based on the sensitivity of Fatty Liver Index (FLI) (57.2%), NAFLD Liver Fat Score (NAFLD LFS) (61.2%), Hepatic Steatosis Index (HSI) (53.5%), Steato Test (43.9%) and Visceral Adiposity Index (VAI) (54.7%) to predict NAFLD as observed in existing literature [12] and with 95% confidence and 20% allowable error, the minimum positive cases come to an average of 24. Using the prevalence of NAFLD (31.2%) the minimum sample size comes to an average of 77.

Data collection

Patients who have MetS were chosen. A detailed history regarding lifestyle diseases, alcohol intake, family history, and medication history was obtained by simple questionnaires. After taking informed consent, physical parameters like waist circumference and vital signs were measured. Blood investigations were sent and Ultrasound Abdomen was done by experienced radiologists. Patients were divided into two groups - those with MASLD and those without MASLD. Blood reports were tabulated, and the five scores were applied to patients in both categories.

Scores used were NAFLD Liver Fat Score, Fatty Liver Index, Steato Test, Visceral Adiposity Index, and Hepatic Steatosis Index [12].

Statistical analysis

Analysis of data was done using the IBM SPSS Statistics 20 Windows (IBM Corp., Armonk, NY, USA). The results are given in mean ± SD or in Median (Q1 - Q3) for all the continuous variables and in frequency (Percentage) for categorical variables. Normality of the data were checked by Kolmogorov Smirnov Z test. The Pearson Chi-Square test was used to find the association between two categorical variables. To test the statistical significant difference in the median level of continuous parameters between patients with and without MASLD, Mann Whitney U test was applied. To test the statistically significant difference in the mean level of continuous parameters between patients with and without MASLD, independent sample t-test was applied. Receiver operator characteristic curve (ROC) analysis was conducted to calculate the area under the curve (AUC) to find the optimal cut-off of five scoring systems (Fatty Liver Index, NAFLD Liver Fat Score, Hepatic Steatosis Test, Steato Test, Visceral Adiposity Index) to predict MASLD and corresponding sensitivity and specificity was estimated. To test the statistical difference in the AUC between two ROC curves, DeLong test statistic was applied. p value of <0.05 was considered statistically significant. All tests of statistical significance were two-tailed.

## Results

Out of 100 patients with MetS, 65% (N =65) had MASLD and 35% (N=35) did not have MASLD. Out of 100 patients, 50% (N=50) were females and 50% (N=50) were males. Seventy-four percent (N=37) of the male samples were MASLD positive and 56% (N=28) of the female samples were MASLD positive. Mean age of patients in the MASLD group was 58 years. The patient characteristics are summarized in Table 1.

**Table 1:** Patient Characteristics + Chi square; t – Independent samples test; u – Mann Whitney; N – Number of patients; % – Percentage; SD – Standard Deviation; Q – Median or 50th percentile; Q1 – 25th percentile; Q3 – 75th percentile. p value < 0.05 is statistically signficant. HOMA-IR: Homeostasis Model for Insulin Resistance, ALT: alanine aminotransferase, AST: aspartate aminotransferase, LDL: low-density lipoprotein, HDL: high-density lipoprotein, MASLD: metabolism dysfunction-associated steatotic liver disease

Variable			Value	Test Statistic	p value
Gender - Female	N (%)	No MASLD	22 (44%)	3.56 ^+^	0.46
		MASLD	28 (56%)		
Age	Mean ± SD	No MASLD	56.23 ± 14.433	0.97 ^t^	0.335
		MASLD	59.09 ± 13.8		
Height	Mean ± SD	No MASLD	1.64 ± 0.089	1.1^ t^	0.217
		MASLD	1.66 ± 0.088		
Weight	Mean ± SD	No MASLD	72.54 ± 10.17	1.1 ^t^	0.236
		MASLD	75.14 ± 10.48		
Body-Mass Index (BMI)	Mean ± SD	No MASLD	26.63 ± 2.41	0.43 ^t^	0.664
		MASLD	26.89 ± 3.1		
Waist circumference	Mean ± SD	No MASLD	90.43 ± 6.72	2.03 ^t^	0.044
		MASLD	92.82 ± 4.87		
Type 2 Diabetes Mellitus	N (%)	No MASLD	14 (28.6%)	1.74 ^+^	0.13
		MASLD	35 (71.4%)		
Systemic Hypertension	N (%)	No MASLD	25 (32.9%)	0.617 ^+^	0.29
		MASLD	51 (67.1%)		
Dyslipidemia	N (%)	No MASLD	21 (32.3%)	0.59 ^+^	0.29
		MASLD	44 (67.7%)		
Hemoglobin	Q(Q1-Q3)	No MASLD	12(11-13)	1068 ^u^	0.606
		MASLD	12(11.5-13)		
Total WBC count	Mean ± SD	No MASLD	7.14 ± 1.75	- 0.466 ^t^	0.642
		MASLD	6.98 ± 1.55		
Platelet	Mean ± SD	No MASLD	251.28 ± 58.27	1.18 ^t^	0.256
		MASLD	269.21 ± 78.23		
CRP	Q(Q1-Q3)	No MASLD	2(0.9-5.5)	433 ^u^	0.59
		MASLD	3(1.03-6.75)		
ESR	Mean ± SD	No MASLD	23.55 ± 17.95	-0.257 ^t^	0.79
		MASLD	24.69 ± 19.79		
Glucose	Q(Q1-Q3)	No MASLD	121(99.7-150)	822 ^u^	0.176
		MASLD	110(88-138.42)		
HOMA-IR	Q(Q1-Q3)	No MASLD	78(52-167)	872 ^u^	0.75
		MASLD	65.2(37.35-112.4)		
HbA1c	Q(Q1-Q3)	No NAFLD	6(6-8)	881.5 ^u^	0.6
		NAFLD	6(6-8)		
AST	Q(Q1-Q3)	No MASLD	19(15.25-25.5)	1025 ^u^	0.41
		MASLD	26(19-32)		
ALT	Q(Q1-Q3)	No MASLD	20(15.75-23)	900 ^u^	0.086
		MASLD	31(20-42)		
HDL	Mean ± SD	No MASLD	46.31 ±13.4	-0.357^ t^	0.72
		MASLD	45.22 ± 15.3		
LDL	Mean ± SD	No MASLD	105.17 ±41.17	0.25 ^t^	0.79
		MASLD	107.31 ±39.06		
Triglyceride	Q(Q1-Q3)	No MASLD	90(70.2-107.5)	686.5^ u^	0.001
		MASLD	111(90-173)		
Total cholesterol	Mean ± SD	No MASLD	173.64± 40.92	-0.136 ^t^	0.8
		MASLD	172.47 ± 40.74		
Insulin	Q(Q1-Q3)	No MASLD	10(7.25-20.75)	956 ^u^	0.189
		MASLD	15(8-27)		
Ferritin	Q(Q1-Q3)	No MASLD	102(73-157)	1089 ^u^	0.729
		MASLD	116(54-279)		
Iron	Mean ± SD	No MASLD	71.83 ±26.68	1.30^ t^	0.196
		MASLD	80.18 ±32.5		
Gamma Glutamyl transferase (GGT)	Q(Q1-Q3)	No MASLD	24(16-32)	1079 ^u^	0.67
		MASLD	24(18-40.5)		
Ceruloplasmin	Mean ± SD	No MASLD	22.06 ±5.07	-0.2 ^t^	0.83
		MASLD	21.83 ±5.4		

Grade one fatty liver (Mild) was present in 80% (N=52), 16.9% (N=11) had Grade two fatty liver (Moderate) and two patients (3%) had Grade three fatty liver (Severe).

Although the number of patients was higher, there was no statistical significance between MASLD and age (p=0.335), Body Mass Index (BMI) (p=0.664), weight (p=0.236) or height (p=0.217). Waist circumference significantly correlated with MASLD with a p value <0.05.

Among patients with MASLD, there were 71.4% (N=35) with type 2 DM and 58.8% (N=30) without type 2 DM, but p value was 0.13 which did not indicate statistical significance. There were 67% (N=51) of patients with systemic hypertension in the MASLD group (p=0.29) and 67% (N=44) of patients with dyslipidemia in the MASLD group (p=0.29). A significant number of patients had elevated levels of Homeostasis Model for Insulin resistance (HOMA-IR) and HbA1c, but there was no statistical correlation between them and MASLD. Triglyceride levels showed statistical significance in comparison to MASLD with p value <0.05.

Eleven percent (N=11) of the entire sample size had normal BMI with MASLD, i.e., Lean MASLD. These data were analyzed and found to have elevated HOMA-IR with alanine aminotransferase (ALT) more than aspartate aminotransferase (AST).

Five scoring indices, namely FLI, NAFLD LFS, VAI, Steato Test and HSI, were compared to determine which could accurately predict MASLD. Area under the curve and p value was calculated, given in Table 2.

**Table 2:** Significance of scores compared to metabolism dysfunction-associated steatotic liver disease (MASLD) p value less than 0.05 is taken as statistical significance.

Scoring Index	Area Under the Curve (AUC)	Standard Error	p value	95% Confidence Interval of AUC	
				Lower Bound	Upper Bound
Fatty liver index (FLI)	0.653	0.058	0.009	0.540	0.766
Hepatic Steatosis Index (HSI)	0.459	0.063	0.218	0.336	0.583
NAFLD Liver Fat Score (NAFLD-LFS)	0.630	0.059	0.030	0.514	0.746
Steato test	0.616	0.057	0.040	0.504	0.728
Visceral Adiposity Index (VAI)	0.628	0.058	0.033	0.514	0.742

**Figure 1:**
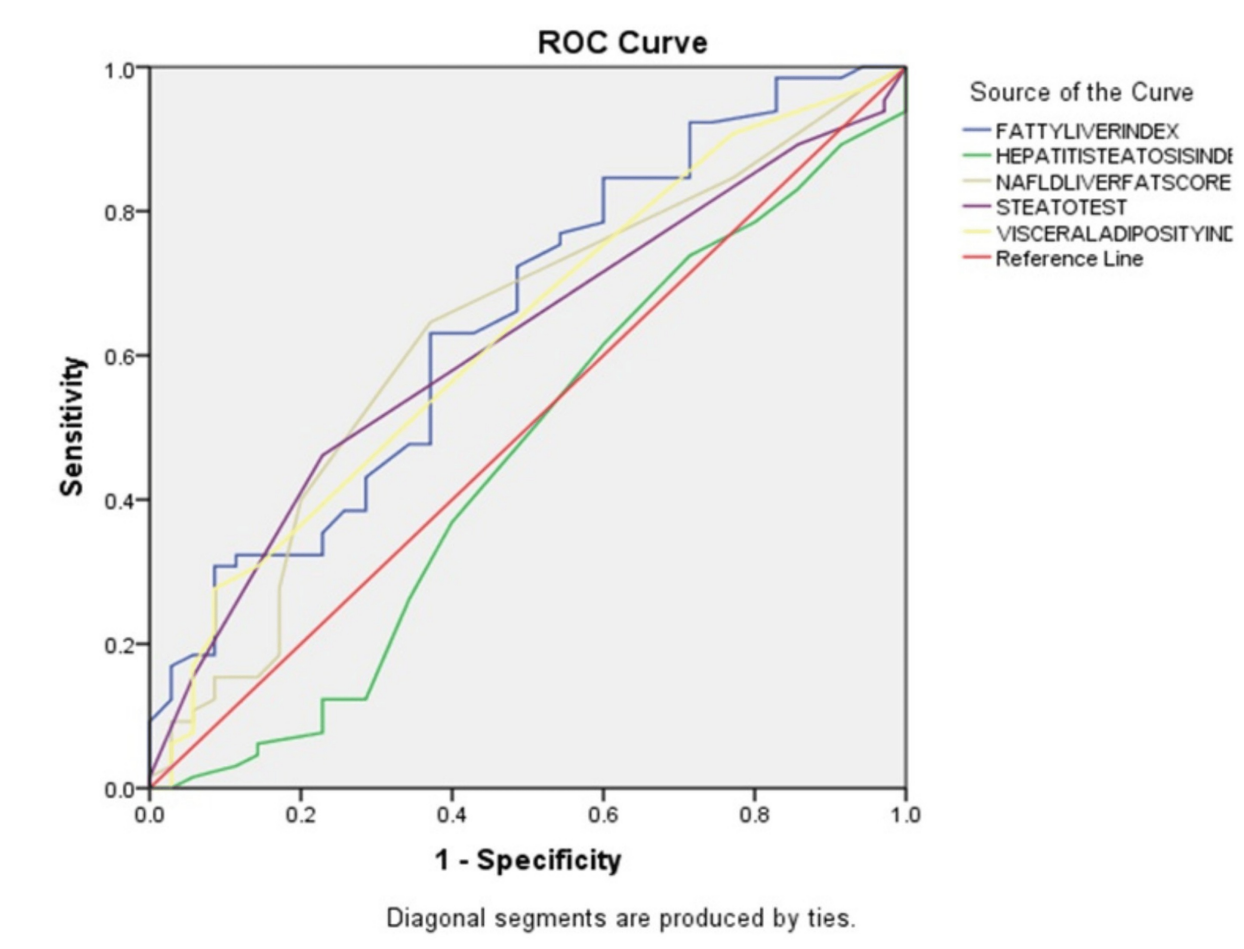
ROC curve for comparison of scoring systems for diagnosis of metabolism dysfunction-associated steatotic liver disease (MASLD) ROC - Receiver Operating Characteristic

Fatty Liver Index, Steato Test, Visceral Adiposity index and NAFLD Liver Fat Score were found to be statistically significant for the prediction of MASLD (with p value <0.05). AUC for Fatty Liver Index was 0.65 and was 0.63 for NAFLD Liver Fat Score. Hepatic Steatosis Index was not found to be a statistically significant predictor. However, there was no statistical curve of different scoring indices (all pair p value shows >0.05). ROC was charted, as in Figure 1.

Cut-off scores for Fatty Liver Index, Steato Test, Visceral Adiposity Index, NAFLD Liver Fat Score and Hepatic Steatosis Index were 42, 4, 4.5, 0.5 and 35.5 respectively. Sensitivity and specificity of the five scoring systems were calculated and showed sensitivity of 64.6% for NAFLD Liver Fat score and specificity of 77% for Steato Test. The scores were not able to differentiate between the different grades of fatty liver (Table 3).

**Table 3:** Sensitivity and Specificity of Scoring Systems NAFLD - Nonalcoholic fatty liver disease

SCORING SYSTEMS	CUTOFF SCORE (POSITIVE IF GREATER THAN OR EQUAL TO) (N)	SENSITIVITY (%)	SPECIFICITY (%)
Fatty liver index	4	63%	62.90%
Hepatic steatosis index	35.5	50.80%	48.60%
NAFLD liver fat score	0.5	64.60%	62.90%
Steato test	4	46.20%	77.10%
Visceral adiposity index	4.5	50.80%	65.70%

## Discussion

Data from 100 patients who had attended the Internal Medicine outpatient clinic were collected, after inclusion and exclusion criteria were applied.

All of the 100 patients (N=100) had MetS, out of which 65% (N=65) had MASLD and 35% (N=35) did not have MASLD.

Out of the 65 samples with MASLD, 74% (N=37) of the samples were male. In a previous systematic review [13], gender differences in various parts of the world were compared. It showed a higher prevalence of MASLD among males than females in Japan, Korea, South China, Pakistan and Spain. However, the same review showed that in India, there was a slightly higher prevalence in females. Most studies showed that men outnumbered women when it came to prevalence of MASLD. In study by Cheng et al. [14], the peak prevalence in males was between ages 41-49.

Majority of the sample population had BMI of more than 25kg/m2, with a mean value of 26.7kg/m2. There were patients with BMI less than 25kg/m2 also, termed Lean MASLD. [15]

The mean waist circumference was 91.9cm with a minimum of 80cms and maximum of 110cms. Waist circumference comparison had a p value <0.05 and is statistically significant for MASLD. According to Qing et al. [16], central obesity is a marker of MASLD. In the study, both BMI (general obesity) and waist circumference (central obesity) were compared. Although both showed independent association, central obesity showed statistical significance, which was similar to our research.

Various scores were calculated based on the values obtained and results were tabulated. Scores calculated included Fatty Liver Index, NAFLD Liver Fat Score, Hepatic Steato Index, Visceral Adiposity Index and Steato Test. Independent samples test for Fatty Liver Index and Hepatitis Steatosis Index showed a significant p value for Fatty Liver Index. Mann Whitney test showed statistical significance for Steato Test, Visceral Adiposity Index and NAFLD LFS. ROC graph showed AUC closest to reference for Fatty Liver Index (0.653). NAFLD LFS had AUC of 0.63, Visceral Adiposity Index had an AUC of 0.62, Steato Test had an AUC of 0.61 and Hepatic Steatosis Index had an AUC of 0.45.

The original study for formulation of Fatty Liver Index by Bedogni et al. postulated that <20 ruled out MASLD and >60 ruled in favor of MASLD [17]. In this study, MASLD was predictable with a value greater than or equal to 42 at 95% CI, Sensitivity of 63% and specificity of 63%, which is similar to the study by Bedgoni et al.

In a study by Liu et al., comparison of the scoring systems in patients with MASLD showed that AUC was maximum for Fatty Liver index followed by NAFLD LFS with only marginal differences [12], which is consistent with this study. There was no significant variation among the mean scores and the grades of fatty liver, hence it cannot be used for differentiating the grades of fatty liver. Independent samples test showed no statistical correlation between the scores and grades of MASLD.

MASLD was predominant in patients with type 2 DM. 71.4% of patients who had type 2 DM also had MASLD. Many studies confirmed that patients with type 2 DM are at higher risk of development of MASLD and routine screening should be done [18]. Thirty-nine percent (N=39) of the total sample size had HbA1c more than 6.5%, i.e. in the diabetic range [19], out of which 22 (56%) had MASLD. Thirty-one percent (N=31) were in the pre-diabetic range (HbA1c 5.7-6.5), out of which 20 (64.5%) had MASLD.

Homeostasis Model for Insulin Resistance or HOMA-IR was calculated. In a study by Gutierrez et al., HOMA-IR of 4.5 was estimated to be an optimal threshold for discriminating MASLD from non-MASLD cases [20]. In a more recent study by Zeng et al., a cut-off of 3.13 was obtained to differentiate MASLD and non-MASLD [21]. In our study, 49% (N=49) of patients had HOMA-IR >3.13, out of which 69% (N=34) had MASLD. However, Mann Whitney test did not show significant statistical correlation between MASLD and HOMA-IR.

A study by Fan et al. showed elevated triglyceride (TG) and low high-density lipoprotein (HDL). TG/HDL was shown to have independent association with MASLD [22]. When comparing separate parameters, HDL, low-density lipoprotein (LDL) and total cholesterol had no statistical significance. But triglyceride levels showed statistical significance in comparison with MASLD, with a p value less than 0.05.

In patients with MASLD, Sanyal et al. have demonstrated elevation of ALT and gamma glutamyl transferase (GGT) in comparison with AST [23]. In our study, there was no statistical correlation between AST, ALT and MASLD. ALT and AST ratio was found to be positively correlating with MASLD, in the study by Zou et al. [24]. In our study, however, it showed no statistical correlation. But it should be noted that, in our study ALT/AST ratio was more than one in half of patients with MASLD, i.e. ALT is more than AST in MASLD patients.

GGT was found to be elevated in 16 (24%) patients out of the 65 patients with MASLD. GGT was found to be frequently elevated in patients with MASLD and was associated with increased mortality [25]. However, there was no statistical correlation between MASLD and GGT in our study.

Eleven percent (N=11) of this study population had Lean MASLD (11 patients with normal BMI and presence of MASLD). The data of these Lean MASLD patients were compared. The number of patients with Lean MASLD were more in men than women. Men who were affected were in a younger age group compared to women. According to Vos et al. [26], there was an increase in Lean MASLD among younger men with a median age of 40 years, which is similar to our study. All of these patients had waist circumference more than 83cm (in women) and 94cm (in men). This shows that even patients with normal BMI, but increased waist circumference, are at risk of development of MASLD. Data from Lean MASLD patients showed that nine patients (81%) had systemic hypertension, more than type 2 DM and dyslipidemia. Eight patients (72.8%) had HOMA-IR greater than 3.13. Although this population is not sufficient to make any concrete evidence, the possibility of Lean MASLD must be kept in mind. Further studies are required for Lean MASLD patients.

This study had a few limitations. It was a single-center study. Ultrasonography was used as the diagnostic marker for fatty liver disease in this study, but the gold standard is liver biopsy, which was not performed due to ethical reasons. Nevertheless, experienced radiologists conducted the ultrasound scans to prevent any errors.

## Conclusions

Fatty Liver Index, NAFLD Liver Fat Score, Visceral Adiposity Index and Steato Test could predict MASLD accurately, with the highest AUC for Fatty Liver Index and NAFLD Liver Fat Score coming marginally close. Hepatic Steatosis Test was not able to sufficiently predict MASLD. However, the scores were not able to differentiate between the grades of fatty liver disease. Although there was no statistical significance, it is worth noting that MASLD was higher in patients with type 2 DM, systemic hypertension and dyslipidemia. In the small sample of patients with Lean MASLD, the number of patients with systemic hypertension was higher. HOMA-IR levels were also elevated in these patients and ALT was found to be more than AST.

Diagnosis of fatty liver disease at the earliest can motivate individuals to make better lifestyle decisions, such as starting a regular exercise program, dietary changes, and appropriately managing their metabolic syndrome. An early change can prevent further progression of the disease.
